# The role of universities in accelerating the sustainable development goals in Europe

**DOI:** 10.1038/s41598-024-65820-9

**Published:** 2024-07-05

**Authors:** Walter Leal Filho, Javier Sierra, Elizabeth Price, João Henrique Paulino Pires Eustachio, Aliaksandr Novikau, Maria Kirrane, Maria Alzira Pimenta Dinis, Amanda Lange Salvia

**Affiliations:** 1 European School of Sustainability Science and Research, Hamburg University of Applied Sciences, Hamburg, Germany; 2 Department of Natural Sciences, Manchester Metropolitan University, Chester Street, Manchester, M1 5GD UK; 3 Research Center for Global Governance; Educational Research Institute: Department of Applied Economics, Faculty of Law, University of Salamanca, Paseo Tomas y Valiente, Salamanca, Spain; 4 Department of International Relations and Public Administration, International University of Sarajevo, Hrasnička Cesta 15, 71210 Sarajevo, Bosnia and Herzegovina; 5 School of Biological, Earth and Environmental Sciences (BEES), University College Cork, Cork, Ireland; 6 Environmental Research Institute (ERI), University College Cork, Lee Road, Cork, Republic of Ireland; 7 Fernando Pessoa Research, Innovation and Development Institute (FP-I3ID), University Fernando Pessoa (UFP), Praça 9 de Abril 349, 4249-004 Porto, Portugal; 8 Marine and Environmental Sciences Centre (MARE), University of Coimbra, Edifício do Patronato, Rua da Matemática, 49, 3004-517 Coimbra, Portugal

**Keywords:** Ecology, Environmental social sciences

## Abstract

The process of implementation of the UN Sustainable Development Goals (SDGs) which were approved by the UN General Assembly in 2015 has not been simple, being influenced by variety of social, economic, and logistical problems. It has also been negatively affected by the COVID-19 pandemic. There are to date no specific studies aimed at assessing the extent to which higher universities institutions in Europe are active in the SDGs implementation process. Departing from this research need, this paper reports on a study aimed at examining the current degree of engagement of European universities in the implementing the SDGs. By using a multi-methods approach, which entails a review of existing documents, a survey involving participants from 22 countries and case studies, the paper maps, documents and disseminates examples of what European universities are doing to implement the SDGs, the challenges they face, and the solutions being deployed to overcome them.

## Introduction

Sustainable development is a concept that has undergone several changes as the years have progressed, and new issues arose. Such evolution has been supported by the participation of various institutions and organisations -including universities- that have been active to implementing the principles and goals of sustainable development. As anthropogenic activity has increased, climate change has progressed, and the resultant effects are being h observed, the need for sustainability is now greater than ever before. The current pressures threaten the survival of humans now, and in future generations. The principle of sustainable development is based on socio-economic development that is in line with ecological/environmental constraints^1^.

The literal definition of sustainable development is any development that can be continued indefinitely. However, the concept has been viewed from several perspectives that have changed the meaning and resulted in numerous definitions. The most cited definition goes back to the Brundtland Report published in 1987, which regards it as “development that meets the needs of current generations without compromising the needs of future generations”. This is further explained as human development that uses resources in a regenerative manner and preserves natural systems^2^. To boost this process, the United Nations has created 17 Sustainable Development Goals (SDGs) that target different aspects of life, especially those that require critical attention and action^3^.

The implementation of the SDGs commenced in 2016 following their introduction in 2015. The framework accounts for development that improved the quality of life until the year 2030. One of the major challenges faced with the SDGs is the implementation of several goals simultaneously without compromising the progress of each goal individually^4^. Researchers struggle with implementing the goals in a coherent manner that has minimal trade-offs^5^.

The 2030 Sustainable Development Agenda aims to tackle social, economic, and environmental (three pillars). Such goals were introduced as an evolution of the Millenium Development Goals (MDGs) and thus incorporated unfinished goals while tackling new challenges from present times. In doing so, global action is promoted where international collaborations are encouraged to ensure that all countries regardless of income status may achieve sustainability^6^. This significantly differs from the MDGs that focused primarily on developing countries. Furthermore, the SDGs cover a broader context since each goal has specific targets to be met while providing proper integration of the three pillars of sustainable development^7^.

The 17 SDGs were introduced as a guidance method to achieve a common goal. In doing so methods have been created to track the progress of the SDGs i.e., measuring and monitoring the achievement of goals. These methods further track practical and theoretical approaches while highlighting the trade-offs visible and finding solutions to minimise them^7^. More specifically, the SDGs call for changes in technology, lifestyle and governance while promoting innovation across all fields. This is an alternative to conventional methods of development that fell short of sustainability in previous times^8^. The goals recognise that eliminating poverty and other social issues must closely be followed with strategies that enhance health and education, diminish inequality, ensure economic growth. This must occur simultaneously with climate change preventing and adaption that preserves the natural environment^9^.

Sustainable Development has featured on the agenda of Higher Education (HE) for more than 20 years^10^, with numerous declarations having been signed by HE leaders^11^ and over 42 national and international networks established dedicated to sustainability in HE^12^. The United Nations (UN) supported initiatives such as the Higher Education Sustainability Initiative (HESI) and the Sustainable Development Solutions Network (SDSN) have been particularly active in promoting the SDGs within higher education^13^.

It is widely accepted that universities play a critical role in delivering the SDGs. Findler et al.^14^ state that Higher Education Institutions (HEIs) have an inherent responsibility to make societies more sustainable. The UN SDSN highlights that role as consisting of four activities, namely, generating knowledge, creating current and future leaders, demonstrating impact, and driving cross-sectoral leadership^15^. Essentially, through increasingly transdisciplinary approaches, universities can build greater connections across the education, research, policy, and practice interface^10^.

University students may learn about the SDGs through both the formal and informal curriculum. Several recent publications have looked at embedding education for sustainable development (ESD) issues as a whole and matters related to the SDGs in particular, into university curricula and the key competencies required by learners to be active in driving forward the sustainability agenda^16^. Informal learning also includes the many student-led initiatives that create “communities of learning” and support interdisciplinary opportunities outside of the often-siloed nature of the formal curriculum^17^. This experiential process can enable students to better apply their learning into the future^18^.

It is increasingly argued that to achieve the ambitious targets of the SDGs, universities need to educate not just our future leaders, but current leaders and decision makers also. Capacity development and professional training opportunities have enormous potential to empower leadership to deliver on the SDGs^10^. In addition, universities can act as “Living Laboratories” supporting co-creation and interdisciplinary solutions-oriented approaches to research and learning. Universities are effectively microcosms of society and therefore can act as a testbed for innovative solutions to global challenges by convening the relevant actors within a “neutral” space^19^. These types of innovative approaches are often more successful at tackling multi-stakeholder “wicked” problems^20^. Findler et al.^14^ document the breadth of ways in which HEIs can impact on SDGs across from direct impacts on research uptake by policy makers to more indirect (and less easily measured) impacts on for example social cohesion and sustainable urban development.

The paper is structured as follows. It starts by explaining the methods used within this research. It then continues by presenting the results and discussing them against the literature. The last section is dedicated to a conclusion, where the main contributions are presented, as well as some limitations and further lines of research.

## Methods

This research used a multi-methods approach, which entails a review of existing literature, an international survey, and an assessment of selected case studies. First, we applied a cross-sectional descriptive analysis of scientific literature on “sustainable development goals” and “higher education” in European countries retrieved from Scopus database. We used Scopus, an online database with around 23,400 available scientific journals across all fields of research^21^. Scopus is frequently used in bibliometric research because it includes a larger number of indexed journals than Web of Science^22^. The current study was conducted on February 18th, 2022, and all data analysis, including citation analysis, was performed on that date. Documents with the words “sustainable development goal$*” or “SDG$” and “universit*” or “higher education” mentioned in the title, abstract, or keywords were retrieved from Scopus for the study period from 2015 (the year in which the United Nations adopted the 2030 Agenda for Sustainable Development) to 2022. The search was limited to documents published by researchers affiliated to European universities, considering books, book chapters, reviews and journal articles, but no language restriction was imposed. The application of this search string allowed us to obtain 638 publications regarding the topic of study. The information retrieved from the Scopus database included complete data regarding citation information, bibliographical information, abstract, keywords, and references. Data in Scopus was transformed into an Excel document for analysis.

For Table 1, which presents key information for the top five scientific peer-reviewed journals in this area, data analysis involved calculating total papers (TP), total citations (TC), citations per paper (CPP), and the SCImago Journal Rank (SJR) for each journal. This analysis allowed the authors to identify the most influential journals in the field and their relative impact based on citation metrics.

**Table 1:** Top five scientific journals.

Journal	TP	TC	CPP	SJR
Sustainability	152	1208	8	0.61 Q1
International Journal of Sustainability in Higher Education	41	228	6	0.73 Q2
Journal of Cleaner Production	14	502	36	1.94 Q1
International Journal of Environmental Research and Public Health	10	11	1	0.75 Q2
International Journal of Management Education	9	166	18	1.17 Q1

Source: Authors elaboration using data from Scoups. TP = total papers; TC = total citations; CPP = citations per paper; SJR = Scimago Journal Ranking.

Table 2, in turn, showcases examples of best practices to integrate the SDGs at European universities, the data was collected through a comprehensive review of initiatives undertaken by various institutions. This included a set of relevant case studies to show some examples of successful initiatives implemented by European universities. For the collection of examples, a table was designed, which entailed a specific set of information, namely the name of the university and country, and the type of SDGs work undertaken. Also, to ensure the tracing of the information, the table contains bibliographical references and weblinks. This also allows a cross-check of the information and enables readers to obtain further details. The case studies were selected aiming to show best practices in different spheres of higher education.

**Table 2:** Some examples of Best Practice to Integrate the SDGs at European Universities.

Example	Aim	University and Country	References
Inter-University Sustainable Development Research Programme (IUSDRP) and Encyclopedia of the UN SDGs	A programme to accelerate the implementation of the UN SDGs and an Encyclopedia to support their implementation	HAW Hamburg, Germany	^47,48^
Carbon Literacy	The first university in the world to teach carbon literacy to its students, training other universities and organisations and sharing a Carbon Literacy for Universities and Colleges Toolkit	Manchester Metropolitan University, United Kingdom	^49^
The Aalborg Model for Problem Based Learning	The Aalborg pedagogical model of problem- and project-based learning, based on real-life issues, directly addresses the UN SDGs. Aalborg publishes the Journal of Problem Based Learning in Higher Education (JPBLHE)	Aalborg University, Denmark	^50^
Green Campus Programme	In March 2020, University College Cork celebrated ten years since becoming the first University in the world to be awarded a Green Flag from the Foundation for Environmental Education	University College Cork, Ireland	^51,52^
Global challenges research	Research addressing global challenges, funded by the Global Challenges Research Fund (GCRF) and the Newton Fund	University of Leeds, United Kingdom	^53^
Learning for Sustainability Scotland	One of the United Nations University Regional Centres of Expertise for Education for Sustainable Development	University of Edinburgh, United Kingdom	^54^
Implementation of Sustainable Development Goals for University Teachers (ImpSDGup)	A teacher training course to guide the integration of ESD in the curriculum of university subjects	University of Girona, Spain	^55^
City-University Partnerships	Contribute to addressing local sustainability challenges and engaging in dialogue about the Sustainable Development Goals	Leuphana University of Lüneburg, Germany	^56^

The authors believe that using the afore-explained literature assessment choices contributed to achieving the paper's goal by providing a comprehensive and structured understanding of the research landscape concerning the implementation of sustainable development goals in higher education. For example, the systematic review and detailed analysis allowed the authors to map out existing research, identify key trends, and highlight significant contributions and gaps. This approach not only facilitated an assessment of the current engagement levels of European universities with the SDGs but also offered insights into the challenges and solutions being deployed, thereby informing future research directions and potential practical implications.

Second, this research used an international survey developed by the research team. The purpose of this questionnaire was to assess the relationship between the SDGs and teaching and research in higher education institutions. For this purpose, the items incorporated in the questionnaire were based on a comprehensive review of the literature regarding the role of universities in fostering sustainability through teaching, research, organizational practices. To ensure the appropriateness of the items included in the survey, a group of specialists provided advice and validated the questionnaire prior to applying it for data collection. This validation process was undertaken by five internationally recognized specialists in the fields of sustainability and higher education. As a result of this validation exercise, the research team adjusted the number of sections and items within each section. Also, some of the elements were adapted to improve their suitability to the research purposes.

After addressing the suggestions made by the experts, and in order to ensure the consistency of the data, a pilot study (i.e. a pre-test) was run with five respondents. The results from this pilot test showed that the survey instrument was adequate, with minor modifications. Both phases (i.e. the validation with specialists and pilot application with additional participants) confirmed and reliability and the validity of the data gathering instrument. The final version of the survey included 22 questions, structured in four sections, namely section 1: Identification (4 items), section 2: Institutional characteristics (3 items), section 3: SDGs and teaching (6 items), and section 4: SDGs and research (9 items).

A Google Form was then developed with the confirmed survey items. A purposive sampling approach, in combination with techniques such as chain referrals and snowballing, was applied to disseminate the link to the on-line questionnaire with a global audience via the research team, the networks of the European School of Sustainability Science and Research (ESSSR), and the Inter-University Sustainable Development Research Programme (IUSDRP). Data collection was carried out from March 2022 to August 2022. Following research ethics protocols, contributors were informed that their participation in the study was voluntary, that their responses would be handled with thorough confidence, and the safety of their personal data will be always guaranteed.

The nature of the research, the methods used, and the fact that no personal data was stored or can be traced back to individuals, conforming with General Data Protection Regulation (GDPR) standards, means that the study is not subject to an ethics permit, as specified by the Association of Medical Ethics Committee in Germany, the body responsible for such assessments in the country leading this study. In any case, and considering any argument requesting waiving consent, all respondents willingly agreed to participate in the study, confirmed through an additional question added to the beginning of the questionnaire, presenting options for yes or no.

Third, this research included a set of relevant case studies to show some examples of successful initiatives implemented by European universities. For the collection of examples, a table was designed, which entailed a specific set of information, namely the name of the university and country, and the type of SDGs work undertaken. Also, to ensure the tracing of the information, the table contains bibliographical references and weblinks. This also allows a cross-check of the information and enables readers to obtain further details. The case studies were selected aiming to show best practices in different spheres of higher education.

### Ethics approval

Experimental protocols did not require institutional approval. Informed consent was obtained from all subjects. All methods were carried out in accordance with relevant guidelines and regulations.

## Results

The research approach applied in this research combines the three methods explained above. This allowed us to explore the state of the art as regards sustainability in higher education by using information from scientific literature, which was completed with the opinion of teachers, researchers, and staff from European HEIs through the survey. Also, we identified successful examples from different HEIs to illustrate how these institutions perform in key areas and what are the reasons for their success. Overall, these results allow us to understand the problems faced by HEIs, and how these barriers could be addressed by policymakers, university administrators, and other stakeholders in promoting sustainability through higher education. Therefore, this multi-method approach improved the quality of the analysis compared to other traditional methods that opt for one of these research strategies only.

### Literature review

**Figure 1:**
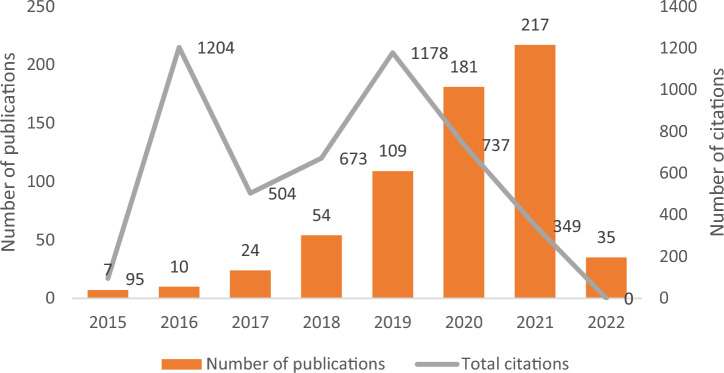
Evolution of publications and citations for the 2015–2022 period. Source: Authors elaboration using data from Scopus.

As regards the literature review, most documents were research articles (n = 425; 67%), followed by conference papers (n = 101; 16%), book chapters (n = 68; 11%), reviews (n = 32; 5%), editorials (n = 7; 1%), and books (n = 3; 0′5%). The database also included one letter and one survey. Increasing interest on the topic of higher education and sustainable development goals is evident, as reflected by the constant growth in the number of publications shown in Fig. 1.

Regarding the top five active journals, the multidisciplinary journal *Sustainability* ranked first (n = 151; 36%), followed by *International Journal of Sustainability in Higher Education* (n = 41; 10%), *Journal of Cleaner Production* (n = 14; 3%), *International Journal of Environmental Research and Public Health* (n = 10; 2%), and *International Journal of Management Education* (n = 9; 2%). Table I shows key information for the top five scientific peer-reviewed journals in this area.

The top five active institutions are the University of Sevilla (n = 19), the Polytechnic University of Valencia (n = 13), the Manchester Metropolitan University (n = 13), the Hamburg University of Applied Sciences (n = 13), and the University College London Institute for Education (n = 12). Regarding institutional affiliation, the majority of papers were published by Spanish researchers (n = 196), followed by scientists from the United Kingdom (n = 143), Germany (n = 67), Italy (n = 42), and France (n = 32). Regarding financial support, the five leading funders are two European Institutions: The European Commission (n = 42) and the European Regional Development Fund (24); two Spanish public administrations: the National Agency for Research (n = 10) and the Ministry of Economy and Competitiveness (n = 8); and a Spanish university: the University of the Basque Country (n = 8).

### Global survey

**Figure 2:**
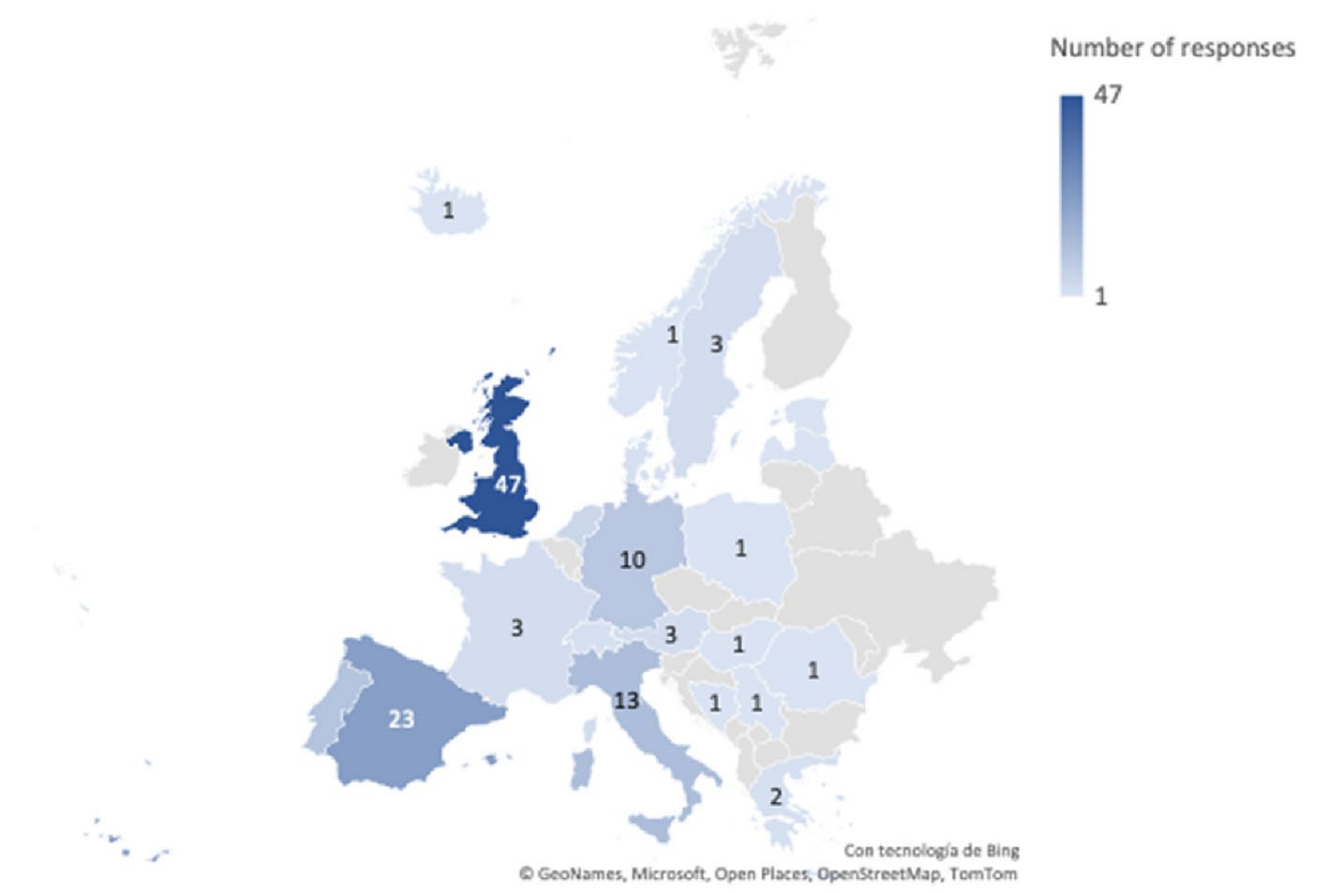
Survey's responses per country.

The second component of this research was an international survey, which was answered by 134 respondents from 22 countries. Regarding their sociodemographic characteristics, there was a gender balance, as reflected by the fact that 49% of participants were female and 50% were male, and one participant declared other. As regards the status of the institution to which they were affiliated, 12% of them work on private higher education institutions, while 88% work on public universities. Figure 2 shows the number of responses collected all over Europe.

**Figure 3:**
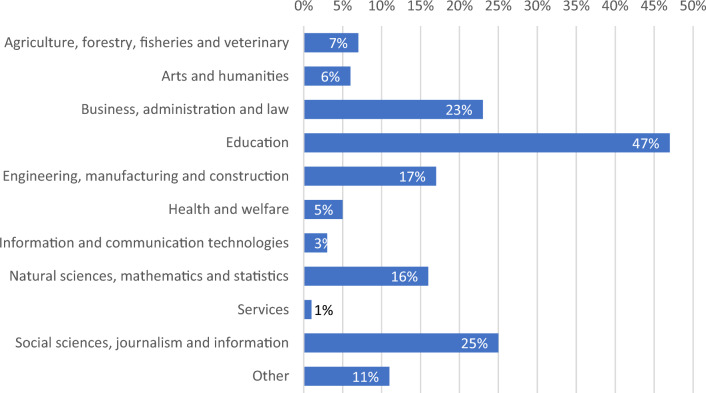
Knowledge area to which participants are affiliated.

Regarding the knowledge area of affiliation, Fig. 3 shows that diverse profiles of educators and researchers answered the survey, even though education and other social sciences-related areas were the most common affiliation among respondents.

**Figure 4:**
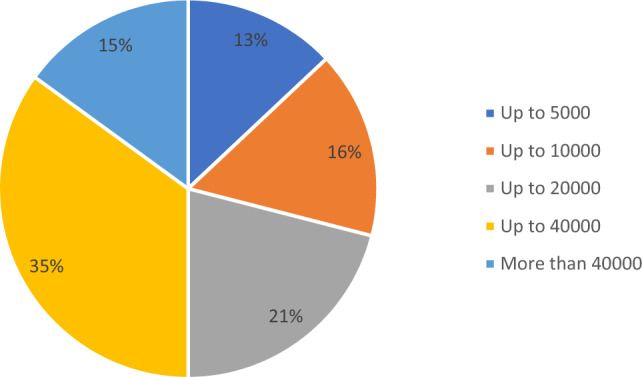
Number of students in the participant’s institutions.

Regarding the size of the institutions in which participants work, the results show a diverse range of HEIs. As reflected in Fig. 4, the most common institutional size is represented by universities of up to 40,000 students (35%), followed by institutions with up to 20,000 students (21%) and up to 10,000 students (16%). The results of the survey showed that the less frequent institutional size are the largest institutions (more than 40,000 students, 15% of the sample) and the smallest ones (up to 5,000 students, 13%).

As regards the question of whether the SDGs were part of their institution´s plans and/or policies, most of respondents declared that these are included in their institution´s plans and/or policies (41%), followed by those who consider that these are included to a little extent (23%), to a great extent (20%), and to a very great extent (12%). Only 4% of respondents stated that the SDGs are not part of their institution´s plans and/or policies at all. In line with this, regarding the question of whether their institutions have SDGs Promoters or Champions, 51% of HEIs recognize this figure or role, while in 49% of institutions this category does not exist.

### Questions regarding SDGs & teaching

**Figure 5:**
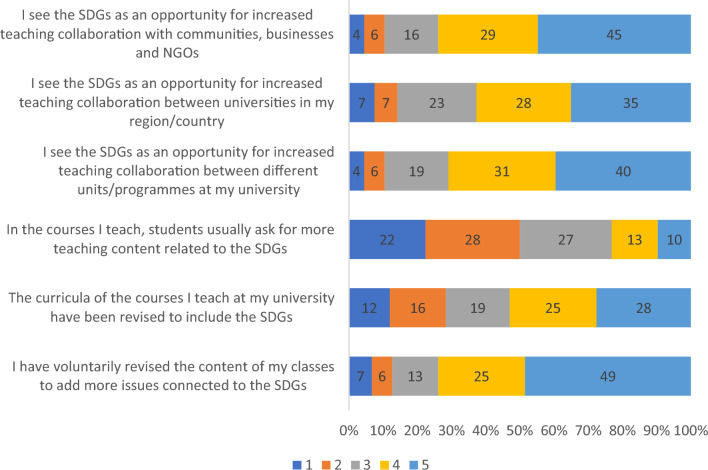
Participants’ level of agreement. Note: Numbers represent percentages. (Scale:1 = Strongly disagree, 2 = Disagree, 3 = Neither agree nor disagree, 4 = Agree, 5 = Strongly agree).

The survey included 6 questions about the connection between the SDGs and teaching. The first question within this category was designed to evaluate the participants’ opinion regarding a set of statements. The results of this question are presented by Fig. 5. Clearly, there is a relatively high consensus (reflected by participants declaring either 4 or 5) as regards most of the statements, except for the case of whether students ask for more teaching content related to the SDGs. Regarding this item, not only the opinions were more diverse, but instead, a relevant number of participants declared that indeed they do not perceive that the students ask for more teaching content connected to the SDGs.

**Figure 6:**
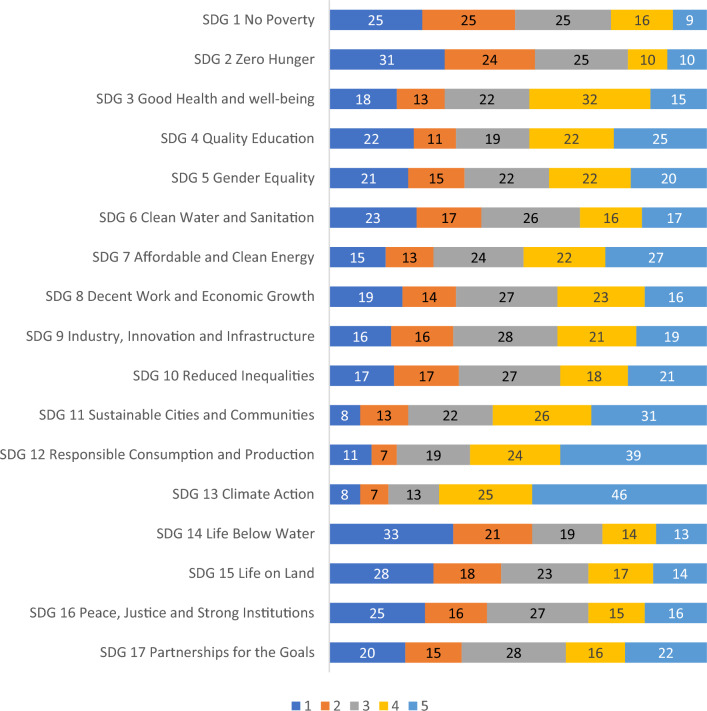
Application of each SDG in teaching. Note: Numbers represent percentages. (Scale: 1 = Not at all, 2 = To a little extent, 3 = To a moderate extent, 4 = To a great extent, 5 = To a very great extent).

The survey also included a question regarding to which extent the participants apply each SDG in their teaching. Figure 6 shows that it is possible to identify three clusters. First, SDGs that are addressed at a large extent in teaching activities (SDGs 11, 12, and 13); second, SDGs that are included at a moderate extent (SDGs 3 to 10, and 17); and third, SDGs that are less frequently addressed (SDGs 1, 2, 14, 15, and 16).

When asked about the best approach to promote teaching about the SDGs in the higher education context, most of participants (68%) declared that SDGs-related content should be distributed across all courses and disciplines. 16% of respondents consider that it should be addressed in a mandatory discipline offered to all courses, 10% suggest that it should be delivered through an optional discipline offered to all courses, while 7% have preferences for other approaches.

In this context, participants were also asked about their perception regarding the support currently offered to academic staff in their institutions to teach about the SDGs. Most of participants declared that support is either poor (39%) or acceptable (34%). In contrast, 9% consider that it is good, and only 2% perceived it as very good. 16% of respondents declared it to be very poor.

Against this background, the survey included a question regarding the challenges for the implementation of the SDGs in their teaching. The most relevant challenge was the lack of connection between the courses and the goals (49%), followed by lack of knowledge in how to properly conduct teaching on the SDGs (42%), lack of support from the administration (39%), lack of materials or resources (28%), and lack of interest or motivation from students (23%). 8% of participants do not identify any challenges, while 8% of them perceive other challenges that were not suggested in the survey, such as lack of time, and lack of proper training. Related to this, we asked participants how they would expect the emphasis given to the SDGs in their teaching to develop until 2030. Most of them (78%) believe that it is likely to increase, 16% consider that it is likely to remain at the same level, and only 5% think that it is likely to decrease.

### Questions regarding research

**Figure 7:**
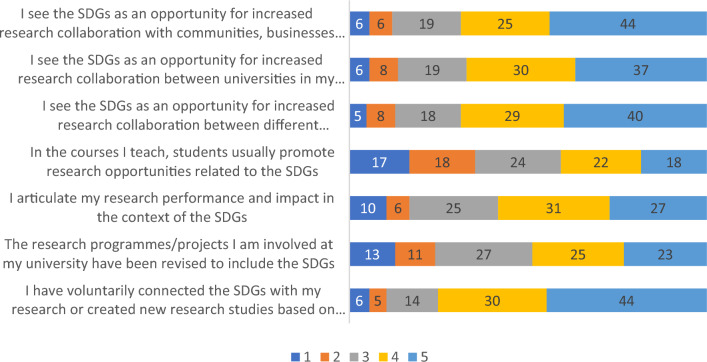
Participants' perception of research opportunities regarding the SDG. Note: Numbers represent percentages. (Scale:1 = Strongly disagree, 2 = Disagree, 3 = Neither agree nor disagree, 4 = Agree, 5 = Strongly agree).

The survey included 9 questions regarding the connection between the SDGs and research. The first question in this section aimed to measure the extent to which participants perceive opportunities for research about the SDGs. Only 126 participants answered to this question, and the results are shown in Fig. 7. Four key areas received a relatively large consensus by participants. The statement for which the largest number of participants reported the highest levels of agreement was “I have voluntarily connected the SDGs with my research or created new research studies based on the goals”, showing the relevant role that the participants’ own interests and motivation play in strengthening research about the SDGs. In line with this, the results show that participants perceive the SDGs as an opportunity for increased research collaborations with different stakeholders. In this context, the most relevant area for this are different programs or units within the participants’ universities, or collaboration with communities, businesses, and NGOs. Next, although with a slightly lower level of support, participants identify the SDGs as an opportunity for increased collaboration with other universities or HEIs.

**Figure 8:**
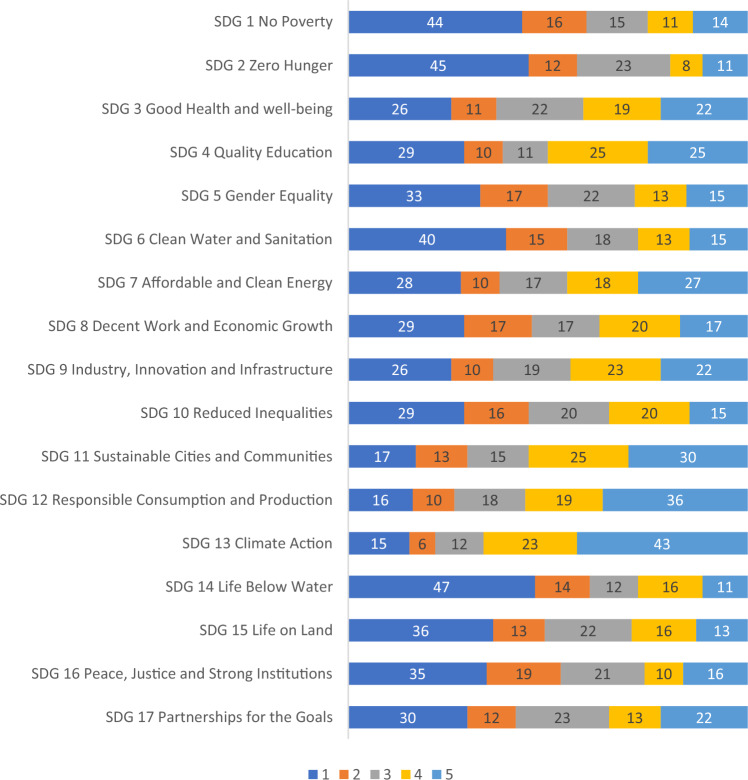
Participants' application of SDGs in research. Note: Numbers represent percentages. (Scale: 1 = Not at all, 2 = To a little extent, 3 = To a moderate extent, 4 = To a great extent, 5 = To a very great extent).

The subsequent questions regarding the connection between the SDGs and research were responded by 124 participants. As regards to what extent respondents apply each SDG in their research, Fig. 8 shows that SDGs 11, 12, and 13 are the ones that a larger number of participants address in their research. Some other SDGs are still relevant, even though less present in research activities undertaken by respondents (SDGs 3, 4, 7, 8, 9, 10, and 17), while SDGs 1, 2, 5, 6, 14, 15, and 16 are the objectives addressed by a smaller number of researchers.

The questionnaire contained a question designed to assess the respondents’ opinion regarding the support currently offered to academic staff to research about the SDGs. In this regard, 44% of respondents consider that it is poor; 29% perceive it as acceptable; 14% declared it to be very poor; 10% think that it is good; and only 4% recognise that it is very good. Against this background, the survey included a question regarding the best approach to promote research about the SDGs in you’re the participants’ education context. 51% of respondents considered that the best option is to distribute it across all research projects or programmes, while 42% of them believe that it may be better to have an institutional centre dedicated to supporting and connecting research on the SDGs. 7% of respondents perceive that the best option is to combine both approaches, or opt for other specific alternatives. To better understand the implications of these perceptions, we asked the participants their opinion as regards the main challenges for the implementation of the SDGs in their research. The main challenge is the lack of support from the administration (48%), followed by lack of connection between research and the SDGs (42%), lack of materials and resources (23%), and lack of interest or motivation from students (17%). 11% of participants do not perceive any challenge, while 10% perceive other reasons, such as for example the lack of central leadership, the lack of funding, and the difficulty to get funding if research is of multi-disciplinary and inter-disciplinary nature. Nevertheless, we also asked the participants about their expectations regarding the emphasis given to the SDGs in their research to develop until 2030, and a large share of them (78%) believe that it is likely to increase. In contrast, 20% consider that it is likely to remain at the same level, while only 2% believe that it is likely to decrease.

**Figure 9:**
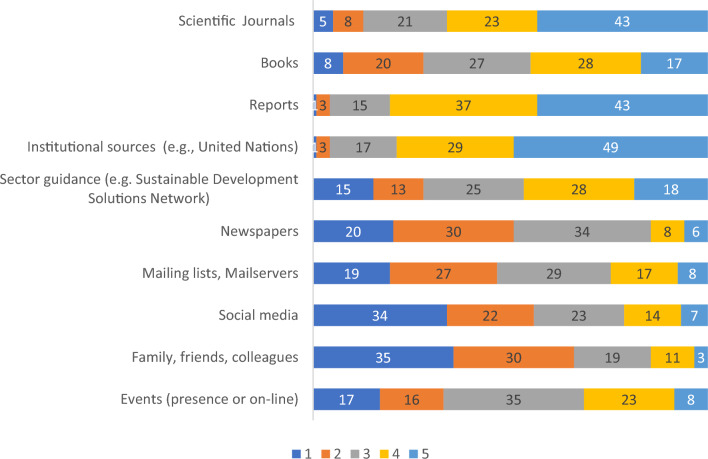
Information sources used to receive information about the SDGs. Note: Numbers represent percentages. (Frequency of use: 1 = not at all, 2 = seldom, 3 = sometimes, 4 = often, 5 = very often).

The survey included a question regarding the main information sources used by participants to receive information about the SDGs. This question was answered by 119 participants, and the results are shown in Fig. 9. It is clear that there are three main sources of information used for gathering information about the SDGs: reports, institutional sources, and scientific journals, followed by books and guidance documents published by relevant stakeholders.

**Figure 10:**
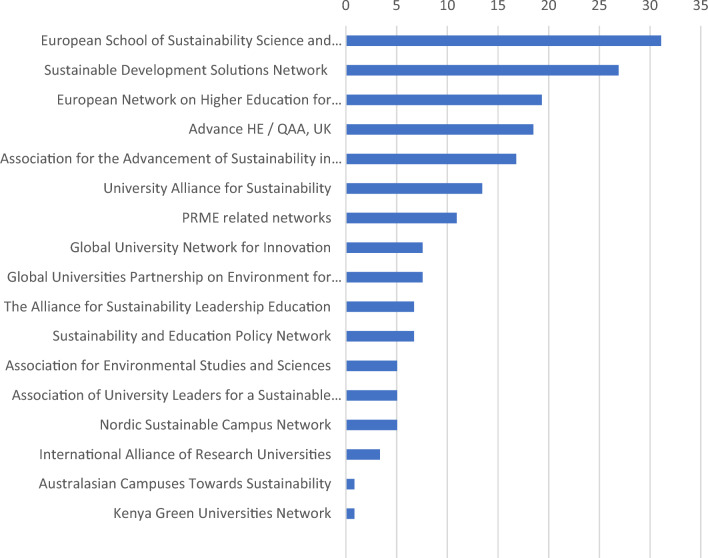
Use of networks to receive information about the SDGs. Note: Numbers represent number of responses.

We also asked participants whether they get information from networks about the SDGs. As shown in Fig. 10, the European School of Sustainability Science and Research is the most popular network for these purposes, followed y the Sustainable Development Solutions Network and the European Network on Higer Education for Sustainable Development.

The survey also included a question regarding to which measures of success does the participants’ institution use to demonstrate the benefit of embedding the SDGs in Higher Education. The Times Higher Impact Rankings is the most popular measure, as declared by 43% of respondents, followed by the EAUC Green Gown Awards (18%), the People and Planet University League, UK (14%), the Sustainability Tracking, Assessment and Rating System (12%), and the United Nations Academic Impact (11%). 25% of participants declared other methods, and 16% stated that their institutions do not use any measure of success.

Finally, we asked participants regarding their opinion about the future use of digitalisation as a tool to teach or undertake teaching and research on the SDGs (e.g., Information and Communication Technologies, Distance Learning). A large share of participants (76%) believe that it is likely to increase, 21% consider that it is likely to remain at the same level, while 3% think that it is likely to decrease.

### Case studies

The last research component included 8 case studies selected to show best practices implemented by European universities in areas such as teaching, research, training, institutional practices, or multi-stakeholder alliances. Regarding research initiatives, the Hamburg University of Applied Sciences (HAW) started an international program to accelerate the implementation of the SDGs, and one of the main axes of this project is the *Encyclopedia of the UN SDGs*, a global effort to boost knowledge about sustainability and the SDGs. On a different standpoint, the University of Leeds has implemented over 160 research projects in more than 30 countries in recent years aiming to foster cross-cutting research, enhance international collaboration and promote economic development, with a strong focus on the Global South. As regards the SDGs and teaching, the Manchester Metropolitan University was the first university in the world that started a program focused on carbon literacy. This program uses peer-to-peer training, and is designed to help their students, other universities, and other relevant stakeholders. From a different perspective, the Aalborg University implemented an innovative pedagogical model that addresses real-life issues using problem- and project-based learning. In line with this, the University of Edinburgh created the Learning for Sustainability Scotland program, recognized by the United Nations as one of its Regional Centres of Expertise for Education for Sustainable Development. Training activities for teachers, researchers and staff are another sphere in which universities are very active. One example of this is the Implementation of Sustainable Development Goals for University Teachers (ImpSDGup) of the University of Girona, designed to help teachers integrate sustainability-related concepts and activities in their teaching. Regarding institutional practices and strategies, the University of Cork was the first university ever to be awarded as a Green Flag by the Foundation for Environmental Education, because of the launch of its Green Campus Program. In parallel to this, alliances between different stakeholders are key for accelerating the implementation of the 2030 Agenda, as stated by SDG 17. One good example of these strategies are the city-university partnerships promoted by the Leuphana University of Lüneburg. Table 2 shows the details of these examples.

## Discussion

The literature review showed that interest in sustainability-related topics has increased exponentially over the last few years. At the same time, it highlighted the relevance of research articles as the main documents addressing these issues. It also revealed the prominence of several scientific journals that several scientific journals that have a clear focus on sustainability or have included sustainability as one of the main criteria they use when making decisions on whether to publish scientific studies. This is in line with the same trends in other areas of knowledge, where sustainability is becoming increasingly relevant for research.

Regarding the survey, the data collected from 134 respondents across 22 countries offers a comprehensive insight into the various facets of SDG implementation in European universities. SDGs are usually viewed as an extremely useful tool for universities to increase cooperation and networking with other organizations – first of all, with other educational institutions and non-governmental organizations to develop new research, facilitate knowledge and technology transfer^23,24^. The absence of network cooperation with external organizations is frequently cited as obstacles to the successful implementation of SDGs within universities^25^. Consequently, European scholars perceive SDGs as an opportunity to enhance teaching and research collaboration between various units and programs within their institutions, as well as with other universities in their region or country, Non-governmental organisations (NGOs), communities, and businesses (Figs. 5, 7).

In order to effectively integrate the principles of SDGs into teaching, it is necessary to develop appropriate curriculum structures and materials^26^. As a result, approximately half of the respondents reported that their course curricula have been revised to incorporate SDGs (Fig. 4). This supports previous findings that universities are still lagging behind in offering courses that fully integrate SDGs into their curricula^27–29^. Studies have shown that the inclusion of SDG principles into curricula is more effective when implemented at a higher level, such as at the academic program, department, or university level^30^. However, most respondents reported that they voluntarily revised the content of their courses without external pressure (Fig. 4). Quite similarly, individual researchers usually voluntarily connect SDGs with their research (Fig. 6).

SDG13 Climate Action is the most widely applied SDG in teaching and research at European universities, as shown in Figs. 5 and 7. Although climate change issues are rarely included in the general education curriculum at universities^31,32^, global warming remains one of the most popular environmental topics and therefore, focus of SDG teaching and research. The next two most applied SDGs in teaching and research at European universities are SDG12 Responsible Consumption and Production and SDG11 Sustainable Cities and Communities, which are often related to economics and management courses.

Most respondents agree with the dominant view that interdisciplinary and transdisciplinary learning settings, which go beyond a single academic course^33–36^ are a better approach to fostering education for sustainable development. However, there is no consensus regarding research on SDGs, specifically whether they should be included in all research programs or concentrated in a single research center.

The survey results confirm previous studies regarding the existence of institutional obstacles to the implementation of SDGs at universities, both in teaching and research. The most significant barriers include poor financial support and a general lack of interest in sustainable development from university administrations and colleagues^25,27,37,38^. However, the reported lack of interest in SDGs from students and their promotion of research opportunities, as shown in Figs. 4 and 6, is a concerning sign that may require changes in teaching approaches of SDGs. Nonetheless, most respondents are optimistic that the role of SDGs in teaching will increase.

The Internet, in general, has become a prevailing source of information about SDGs for many, including educators^39^. Although social media can be effective for dissemination SDG-related information towards students and general public^40^ educational professionals in Europe primarily rely on academic sources such as peer-reviewed articles, official reports and institutional sources (Fig. 8).

Participation in cross-institutional networks, initiatives, and alliances is important for implementation of SDGs in universities and explicitly encouraged in SDG17 Partnerships for the Goals^41^. Although European scholars receive information about SDGs from many networks, two of the them are the most popular (Fig. 9)—European School of Sustainability Science and Research^42^ and Sustainable Development Solutions Network^15^.

University rankings provide a systematic approach to evaluating the performance of higher education institutions. However, among existing rankings, those that value sustainability are scarce^43,44^. The Times Higher Education Impact Rankings, one of the few academic rankings that assess universities’ performance in sustainable development, is currently considered the most popular international ranking system for measuring the integration of SDGs in universities.

Numerous studies have demonstrated the increasing role of digitalization, including e-learning, in teaching SDGs^41,45,46^. Similarly, respondents from European universities have expressed their belief that the use of digitalization as a tool for teaching and conducting research on SDGs will continue to grow.

From the strict point of view of HEI’s internal management and operations, these results point out the need to increase cooperation among different departments, services, and units within the institution, and to increase cooperation with other HEIs from different contexts. To accomplish this, it may be useful to implement an internal survey to gather information on this topic among all levels of the university community. This will also allow the identification of priorities, bottlenecks, and objectives among teachers, researchers, and staff. Also, these results suggest that it becomes crucial for HEIs to create and promote networks for enhancing cooperation with other stakeholders from outside academia, both from the public and private sectors. This will help HEIs to improve the implementation of the SDGs internally, but at the same time will contribute to strengthening the role that HEIs can play in fostering synergies with other actors out of university campuses.

As regards teaching and learning activities, we identified that, even though around half of the participants in the survey declared that they have updated their curricula to include SDG-related content, these results show that there is still a large room for improvement. Therefore, HEIs could develop training programs and provide guidance to support researchers, teachers, and staff in updating study plans and syllabuses under the umbrella of the 2030 Agenda. In line with this, it may be wise to develop mechanisms to foster interdisciplinary and transdisciplinary learning settings. This may require updating study plans and increasing flexibility and creativity for course recognition among different degrees and faculties. To do so, implementing faculty-wide or campus-wide courses could be a powerful approach.

Finally, the analysis of the case studies offered some insights into several successful experiences executed by HEIs in spheres such as teaching, research, training, institutional practices, or multi-stakeholder alliances. These initiatives prove that universities and other HEIs can foster the achievement of the SDGs and, hence, accelerate the implementation of the 2030 Agenda. As explained, this could be done by promoting research; enhancing teaching and learning for ESD; applying science in diverse projects; increasing cooperation with public and private stakeholders; fostering the development of international, inter-disciplinary, and multi-disciplinary networks; creating research centers and institute with a focus on sustainability; and providing training and support for teachers, researchers, and staff. Overall, these examples show the manyfold approaches that HEIs could adopt to promote sustainability at several levels and the pivotal role that they can play in enhancing synergies with different stakeholders and economic sectors.

## Conclusions

This paper reports on a study aimed at examining the current degree of engagement of European universities in the implementing the SDGs. It has used a multi-methods approach, consisted of a review of existing documents, a survey involving participants from 22 countries and case studies.

The remit of the paper is to map, document and disseminates examples of what European universities are doing to implement the SDGs, the challenges they face, and the solutions being deployed to overcome them. From the evidence gathered from the paper, some main conclusions can be made. The first, is that it is encouraging to see that the number of institutions which do not seem to take the SDGs into account is rather small, and that over 30% of the sample currently handle the SDGs to a great or very great extent. This is an encouraging sign. The second conclusion which can be drawn relates to the fact that the emphasis given to the SDGs in teaching does very, and some SDGs (e.g., SDGs 1, 2, 14, 15, and 16) seem to be less frequently addressed. Also, the study has shown that some SDGs are quite present as far as research is concerned (i.e., SDGs 11, 12, and 13), whereas others (SDGs 1, 2, 5, 6, 14, 15, and 16) are being addressed by a smaller number of researchers. The final conclusion from the paper is that less than 15% of the sample stated they received institutional support to the SDGs. This is a worrying trend, since lack of support is known to be an obstacle for engagement (Leal Filho et al. 2023).

The paper has some limitations. One of them is the fact that the review of the documents focused on the SDGs and not on general sustainability issues. Also, the survey sample, with just over 100 respondents cannot be regarded as very comprehensive. Moreover, the case studies focused on what European universities are doing and did not investigate practices in other geographical regions.

Future studies may focus on what universities in other geographical regions (e.g., The Americas, Africa and Middle East or the Asia–Pacific region) are doing. They may also investigate the existing barriers to the integration of the SDGs in university study programmes. This may be helpful in building a profile of the extent to which universities around the world are engaged in the delivery of the SDGs, and in identifying the means to accelerate this delivery.

## Data Availability

All data generated or analysed during this study are included in this published article.
